# Magnetic Milligripper Platform for Biomedical and Biological Applications

**DOI:** 10.3390/mi17080926

**Published:** 2026-07-31

**Authors:** Doha Abdelrahman, Alain Savary, Bernard Feuillard, Marc Heuschkel, Dario Principi, Adrien Roux, Christophe Besson

**Affiliations:** 1 School of Business and Engineering Vaud (HEIG-VD), University of Applied Science of Western Switzerland (HES-SO), 1401 Yverdon-les-Bains, Switzerland; doha.abdelrahman@heig-vd.ch (D.A.); alain.savary@heig-vd.ch (A.S.); bernard.feuillard@heig-vd.ch (B.F.); 2 School of Engineering, Architecture and Landscape of Geneva (HEPIA), University of Applied Sciences and Arts Western Switzerland (HES-SO), 1202 Geneva, Switzerland; marc.heuschkel@hesge.ch (M.H.); dario.principi@hesge.ch (D.P.); adrien.roux@hesge.ch (A.R.)

**Keywords:** untethered milligripper, magnetic microrobot, electromagnetic actuation, miniature gripper

## Abstract

Achieving precise, untethered manipulation at the millimeter scale remains a fundamental challenge in minimally invasive medicine. Magnetic milligrippers have emerged as promising untethered tools for grasping, transporting, and releasing objects in confined anatomical environments, yet most existing rigid designs rely on multi-component assemblies with dedicated hinges or joints that require complex fabrication processes. Here, we present a simple, cost-effective rigid magnetic milligripper based on a folded titanium structure with two inclined permanent magnets. Actuated by a three-axis Helmholtz–Maxwell coil system, it enables orientation and translation control, as well as reversible opening. The actuation platform is coupled to a joystick-based control interface, allowing intuitive, real-time steering and opening of the milligripper by a single operator. The design is established with an analytical magnetic dipole model and validated through both finite-element simulations and experimental characterization, which together confirm reproducible, fully elastic operation across the investigated actuation range. The strong agreement between analytical, numerical, and experimental results establishes this architecture as a mechanically robust and scalable proof-of-concept platform for magnetic micromanipulation, with direct relevance to future minimally invasive biomedical applications.

## 1. Introduction

Over the past two decades, microrobotics has emerged as a rapidly expanding field, evolving from early chemically powered nanomotors into sophisticated untethered microtools capable of locomotion, targeted delivery, and active manipulation [1,2,3,4,5,6,7]. Additionally, the growing maturity of this field has opened new perspectives for minimally invasive medicine, where such tools offer the ability to navigate confined anatomical spaces and perform complex manipulation tasks with precision and reliability inaccessible to conventional instruments [8,9]. Among the various actuation strategies explored, magnetic control has consistently stood out as particularly attractive for biomedical applications [3,10,11]. External magnetic fields can penetrate biological tissue with minimal attenuation and, at the low static field strengths required for actuation, are considered to pose no known biological harm [3]. Indeed, the recommended maximum static field strength for medical procedures under supervision is 4 T [3], far exceeding the tens of millitesla typically employed in magnetic actuation systems [9,12]. These properties enable fast, fully wireless control of untethered devices across a range of scales [3]. They have also driven the development of increasingly capable magnetic manipulation platforms [13], ranging from static or moving electromagnet systems [14] to fully integrated three-dimensional Helmholtz–Maxwell coil systems [15,16], which now provide the infrastructure for the precise spatial control of microtools in fluidic and biological environments [9,14,17].

Recent advances demonstrate how rapidly laboratory-scale concepts are approaching clinical relevance: clinically ready magnetic microrobots have been reported for targeted therapies in vivo [9], and portable human-scale navigation systems such as the Navion platform enable magnetic catheters to be steered through demanding endovascular trajectories, including deep navigation within the circle of Willis [10]. Within this broader context, gripping devices—milligrippers—represent a distinct category of magnetically actuated microtools. Unlike microswimmers or drug-carrying vehicles, which primarily operate in transit, a milligripper must interact mechanically with its environment by grasping tissue [18], retrieving objects [19,20], or delivering payloads with spatial precision [21]. Such devices enable the controlled grasping, transport, and release of micro-objects in fluidic environments, with direct applicability to targeted drug delivery [21,22] and tissue biopsy in hard-to-reach regions, as demonstrated by magnetically actuated capsule endoscopes retrieving tissue samples from the gastrointestinal tract [18]. A natural extension is catheter-based deployment, which is relevant for vascular, gastrointestinal, and urological interventions. Recent advances in magnetically steered catheters have demonstrated active navigation through micro- and millimeter-scale arteries in vivo [23,24,25], in situ biomechanical force sensing during transluminal procedures [26], and minimally invasive in vivo bioprinting [27], establishing the navigation infrastructure on which milligripper deployment can build. Indeed, the feasibility of combined navigation and grasping has already been demonstrated by integrating a milligripper at the distal tip of a magnetically steered catheter [28].

The development of functional milligrippers has followed its own design trajectory, with actuation mechanisms spanning electrostatic, electrothermal, electromagnetic, optical, and chemical approaches. Early designs relied on chemical stimuli or self-folding mechanisms enabling rudimentary pick-and-place operations but offering little real-time controllability once deployed [19]. Subsequent generations of thermally or light-responsive soft grippers introduced programmable deformation [29]. In contrast, hybrid approaches combining magnetic actuation with stimuli-responsive hydrogels have enabled both locomotion and cargo release in response to local chemical cues [30], while light and magnetic field combinations applied to hybrid hydrogel–metal structures have demonstrated fast and programmable actuation with cargo delivery capabilities [31]. Additionally, soft magnetic microrobots employing NdFeB-loaded elastomeric films actuated by Helmholtz–Maxwell coil systems have been shown to grasp and transport biological cells in fluidic environments [32]. Beyond soft film-based designs, modular approaches using permanent-magnet hydrogels with Janus particles have further enabled self-assembling microrobot chains with four locomotion modes and programmable multi-cargo release, as demonstrated in ex vivo intestinal and cartilage environments [33]. A three-dimensionally programmable untethered milligripper capable of grasping and assembly tasks was demonstrated [20], opening the door to devices that can be steered, oriented, and actuated using a single external magnetic field system. This was followed by demonstrations of shape-morphing and multimodal locomotion in soft magnetic bodies [34], such as magnetically actuated micro-forceps and scissors for minimally invasive neurosurgery [35], multi-magnet gripper architectures driven by both uniform and gradient magnetic fields [21,22], and electropermanent magnet configurations enabling switchable gripping modes that decouple holding force from continuous power input [36]. Untethered microrobots with integrated gripping mechanisms have further been demonstrated for on-chip cell surgery under magnetic actuation [37], high-throughput microparticle gripping across a wide size range in microfluidic environments [38], and gripping in both dry and aqueous environments using miniature soft electromagnetic actuators [39]. Additionally, advanced control strategies incorporating dynamic obstacle avoidance and finite-state machine control have further been demonstrated in soft magnetic milligrippers operating in complex environments [12]. Cost-effective fabrication approaches have also been explored, with helical miniature robots fabricated via needle scribing on polymer substrates demonstrating five distinct locomotion modes under external magnetic actuation, enabling thrombus removal and targeted drug delivery in phantom vascular and gastric environments [40]. Complementary advances in hybrid electromagnetic–permanent magnet actuation systems have further enabled the decoupled control of locomotion, orientation, and morphological deformation in ferrofluidic robots, thus extending their operational range to gastric phantoms and passive payload delivery [41]. A 3D-printed triple-finger magnetic milligripper has also demonstrated robust manipulation of microscale objects using a single electromagnetic coil [42]. Beyond magnetic actuation, force-sensitive compliant grippers with vision-based force and torque estimation have also been demonstrated for closed-loop gripping force control [43], highlighting sensing as an emerging capability across gripper architectures. Despite this progress, a persistent challenge remains: achieving simultaneous controllability of orientation, translation, and gripping while maintaining structural simplicity and mechanical robustness.

In this work, we present a rigid, untethered magnetic milligripper based on a folded titanium (Ti–6Al–4V) structure with two integrated NdFeB permanent magnets, thus enabling orientation, translation, and reversible opening and closing under externally applied magnetic fields. The proposed design relies on magnetic torque-induced elastic bending rather than articulated joints or soft actuating materials, providing a simple, robust, and scalable architecture. A key element of this study is the experimental actuation platform, which combines Helmholtz and Maxwell coils to generate both uniform magnetic fields and controlled magnetic field gradients, thereby allowing the milligripper to be manipulated along three dimensions. Section 2 presents the milligripper design and the theoretical framework governing its actuation, while Section 3 describes the fabrication process and experimental setup. Then, Section 4 reports the experimental results and model validation, followed by a summary of the main findings and perspectives for future work in Section 5.

## 2. Milligripper Design and Theoretical Framework

The design of the magnetic milligripper and the theoretical framework governing its actuation are presented in this section. Specifically, the device geometry and magnet configuration are first introduced, followed by a dipole-based analytical model describing the magnetic forces and torques acting on the system. These relations form the basis for predicting the orientation, translation, and opening behavior of the milligripper under external magnetic fields.

### 2.1. Magnetic Dipole Model

**Figure 1:**
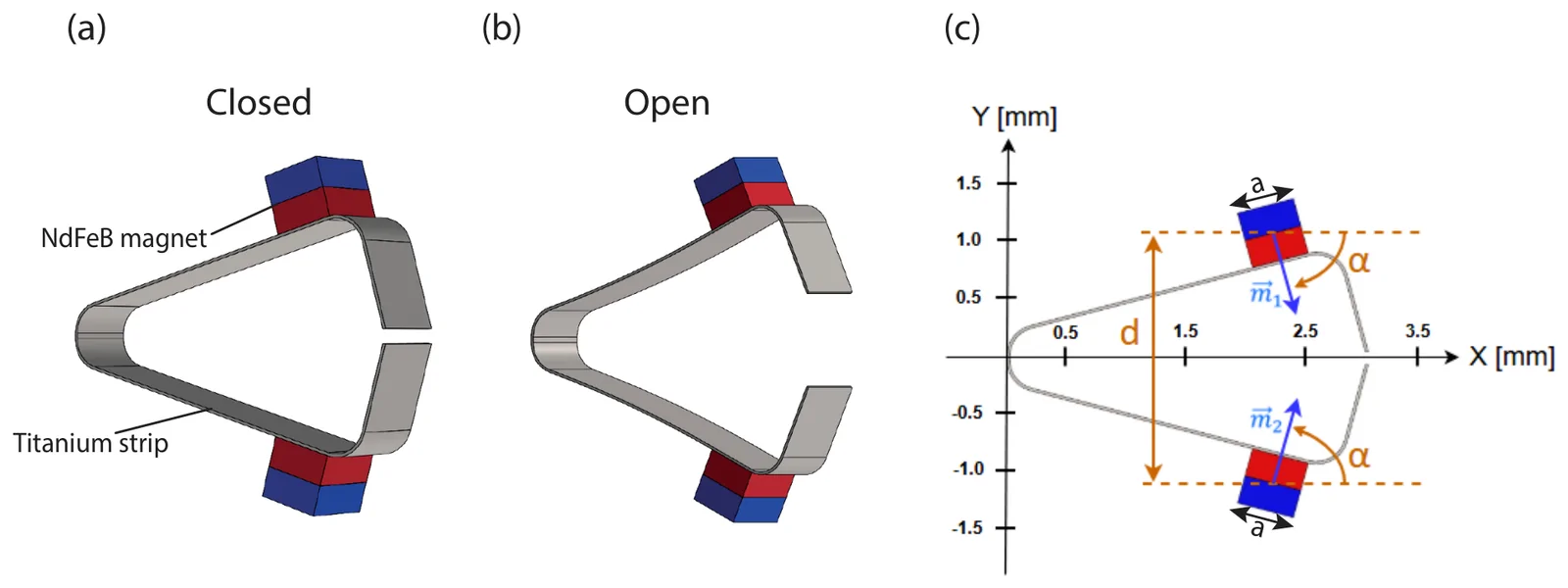
A 3D-rendered model of the magnetic milligripper in its two operating states. (**a**) Closed configuration and (**b**) open configuration. (**c**) Dimensioned schematic of the milligripper geometry in the Oxy plane showing the key structural parameters: magnet side length a=500 µm, inter-magnet center-to-center separation d=2.22 mm, and magnet inclination angle $\alpha =75^{\circ }$ relative to the *x*-axis. The magnetic moment vectors ${\vec{m}}_{1}$ and ${\vec{m}}_{2}$ of the two symmetrically inclined magnets are indicated by blue arrows.

The milligripper consists of a folded titanium strip (Ti–6Al–4V, 8.0×0.5×0.03 mm^3^) with two bonded 500 μm NdFeB (N50H grade) cubic permanent magnets located symmetrically on each arm as shown in Figure 1a,b. In its final assembled configuration, the milligripper fits within an envelope of approximately 3×2.8×0.5 mm^3^, as illustrated in Figure 1c. In the closed configuration (Figure 1a), the two arms converge at the tip and are held together by the elastic restoring force of the titanium structure. When an external field is applied, the arms deflect outward under magnetic torque-induced elastic bending (Figure 1b). Each magnet is modeled as a uniformly magnetized rigid body and approximated as a point magnetic dipole with a magnetic moment.

(1) m=MV

where *M* is the magnetization and $V=a^{3}$ is the magnet volume for a cubic magnet of side length *a*. For NdFeB magnets, the magnetization is related to the remanent flux density $B_{r}$ through

(2) $$M=\frac{B_{r}}{\mu_{0}}$$

where $\mu_{0}$ is the magnetic permeability of the free space.

The two dipoles are symmetrically inclined by an angle ±α (Figure 1c) with respect to the x-axis in the Oxy plane. This inclined configuration is fundamental for enabling orientation, translation, and opening actuation. The distance between their centers is denoted as *d*, as shown in Figure 1c. Additionally, the titanium strip is considered non-magnetic with relative permeability close to unity and therefore does not perturb the magnetic field distribution.

#### 2.1.1. Magnetic Torque for Orientation Under an External Field

**Figure 2:**
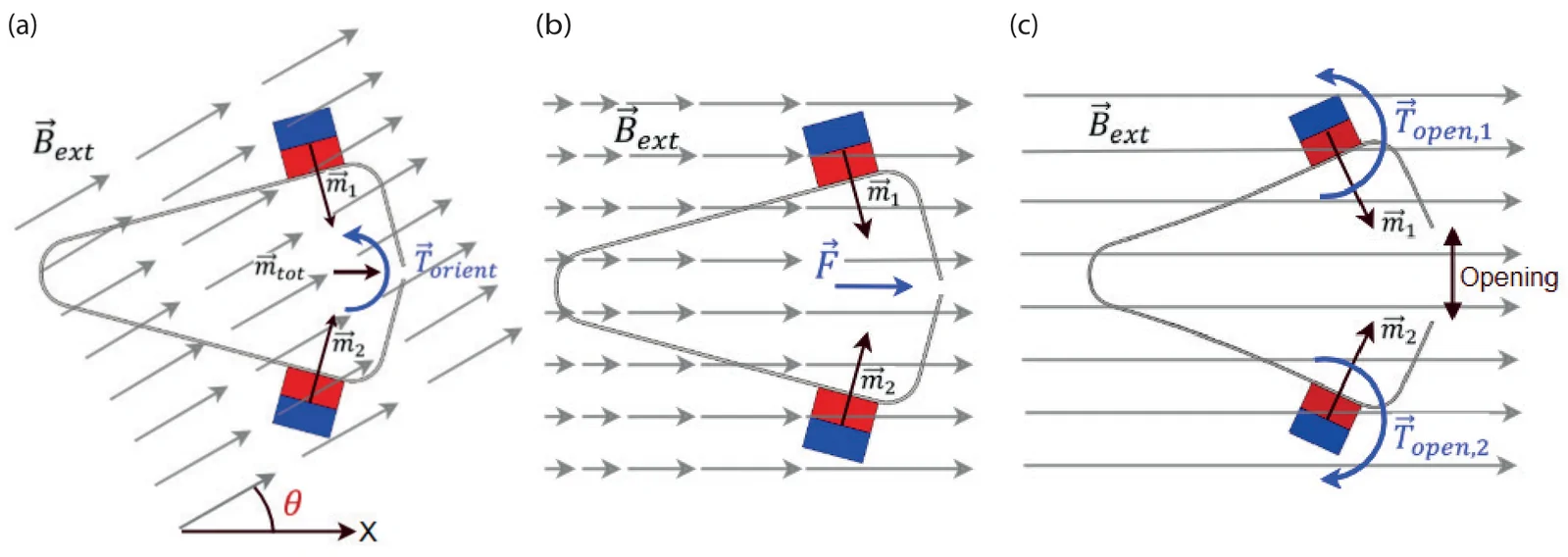
Actuation principle of the magnetic milligripper under an externally applied magnetic flux density ${\vec{B}}_{\mathrm{ext}}$. (**a**) Orientation mode: The total magnetic moment ${\vec{m}}_{\mathrm{tot}}$ of the two-magnet system aligns with the applied uniform field generated by the Helmholtz coils, producing a net orientation torque ${\vec{T}}_{\mathrm{orient}}$ that rotates the gripper in space. (**b**) Translation mode: A magnetic field gradient generated by the Maxwell coils produces a net force $\vec{F}$ on the total magnetic moment, displacing the milligripper within the workspace. (**c**) Opening mode: Individual magnetic torques $T_{\mathrm{open},1}$ and $T_{\mathrm{open},2}$ act on each inclined magnet under the applied field, generating opposing bending moments on the two arms and producing symmetric elastic opening.

Figure 2 illustrates the three actuation modes of the magnetic milligripper under an externally applied magnetic field. When placed in a homogeneous external magnetic field generated by Helmholtz coils (Figure 2a), the milligripper experiences a magnetic torque acting on the total magnetic moment of the gripper:

(3) $${\vec{T}}_{\mathrm{orient}}={\vec{m}}_{\mathrm{tot}}\times {\vec{B}}_{\mathrm{ext}}$$

where ${\vec{B}}_{\mathrm{ext}}$ is the external applied magnetic flux density, ${\vec{m}}_{1}$ and ${\vec{m}}_{2}$ are the magnetic moments of each magnet, and ${\vec{m}}_{\mathrm{tot}}={\vec{m}}_{1}+{\vec{m}}_{2}$. The magnitude of the orientation torque is

(4) $$T_{\mathrm{orient}}=m_{\mathrm{tot}}\;B_{\mathrm{ext}}\sin\theta$$

where θ is the angle between ${\vec{m}}_{\mathrm{tot}}$ and ${\vec{B}}_{\mathrm{ext}}$. This torque aligns the milligripper with the applied magnetic field and enables controlled orientation in space. Since the magnetic moment scales with the magnet volume $a^{3}$, the orientation torque scales with the cube of the magnet size and linearly with the external field amplitude. Therefore, this relationship guided the selection of the magnet dimensions to ensure sufficient torque for reliable alignment.

#### 2.1.2. Magnetic Torque for Opening Under an External Field

When the gripper is aligned within the uniform external field generated by the Helmholtz coils, it remains stationary. However, increasing the external magnetic field induces magnetic torques on the two magnets (Figure 2c), causing the gripper to open through mechanical deformation. When the field is removed, the gripper closes naturally, returning to its original shape. The strength of the external field controls the opening degree of the gripper.

Milligripper opening is driven by the torque exerted individually on each inclined magnet when an external magnetic field is applied along the direction of the total magnetic moment ${\vec{m}}_{\mathrm{tot}}$. In this configuration, $\theta =0^{\circ }$; since the field is homogeneous, the uniform external field produces no net torque on the gripper as a whole and no translational force. However, because each magnet is individually inclined at ±α (Figure 1c) relative to ${\vec{m}}_{\mathrm{tot}}$, the field generates local torque on each arm, producing symmetric elastic bending and opening the gripper. For a single magnet, the magnetic torque is

(5) $${\vec{T}}_{\mathrm{open},i}={\vec{m}}_{i}\times {\vec{B}}_{\mathrm{ext}}$$

where ${\vec{m}}_{i}$ is the magnetic moment of a single magnet, while the magnitude of the opening torque is

(6) $$T_{\mathrm{open},i}=m_{i}\;B_{\mathrm{ext}}\sin\alpha$$

The effective opening torque scales with sinα. Therefore, the opening actuation mechanism is critically dependent on the geometric inclination of the magnets.

#### 2.1.3. Magnetic Force Under an External Field Gradient

When subjected to a magnetic field gradient generated by the Maxwell coils, the milligripper experiences a translational magnetic force (Figure 2b). For a magnetic dipole in a non-uniform magnetic field, the force is given by

(7) $$\vec{F}=\nabla \;\left({\vec{m}}_{\mathrm{tot}}\;\cdot \;{\vec{B}}_{\mathrm{ext}}\right)$$

Because the two magnets are symmetrically inclined by ±α, the vector sum of the two dipoles yields

(8) $$m_{\mathrm{tot}}=2m\cos\alpha$$

where *m* is the magnetic moment of a single magnet. This relationship demonstrates that, while α is between $0^{\circ }$ and $90^{\circ }$, the effective dipole moment available for translational actuation decreases as α increases. Consequently, when the total magnetic moment is aligned along the x-axis, as shown in Figure 2b, the magnitude of the magnetic force generated by a gradient field along the x-axis becomes

(9) $$F_{x}\approx 2m\cos\alpha \;\frac{\partial B_{x}}{\partial x}$$

The translational force, therefore, scales with both magnet volume ($a^{3}$) and the cosine of the inclination angle. While a large α enhances opening torque efficiency, it simultaneously reduces the effective dipole moment contributing to translational motion.

#### 2.1.4. Internal Magnet–Magnet Interaction Without an External Field

Even in the absence of an external magnetic field, the two permanent magnets interact through dipole–dipole coupling. This interaction generates both a magnetic force and a magnetic torque that influence the gripper’s equilibrium configuration.

The repulsive force along the separation direction can be expressed as

(10) $$F_{y}\left(d,\alpha \right)=\frac{3\;B_{r}^{2}\;a^{6}}{4\pi \mu_{0}\;d^{4}}\;\left(1+\sin^{2}\;\alpha \right)$$

where *d* is the center-to-center distance between the magnets (see Figure 1c). Additionally, the magnetic torque acting on one dipole due to the magnetic field generated by the other dipole is

(11) $$T_{12}\left(d,\alpha \right)=\frac{B_{r}^{2}\;a^{6}}{8\pi \mu_{0}\;d^{3}}\sin\left(2\alpha \right)$$

These internal interactions tend to promote gripper opening. Additionally, their magnitude decreases rapidly with distance ($∼d^{-3}$ and $∼d^{-4}$), ensuring that magnet coupling weakens as the arms separate. The internal torque also depends strongly on α.

### 2.2. Magnetic Field Generation

A pair of Helmholtz coils generates a uniform magnetic field at the center of the coil system. For coils with radius *R* and *N* turns carrying current *I*, the externally applied magnetic flux density at the center is given by [16]

(12) $$B_{\mathrm{ext}}=\frac{8}{5\sqrt{5}}\;\frac{\mu_{0}\;N\;I}{R}$$

This magnetic flux density $B_{\mathrm{ext}}$ is used in the analytical torque expressions. Similarly, a pair of Maxwell coils generates a magnetic flux density gradient used for translational actuation, with the magnetic field gradient along the x-axis expressed as [16]

(13) $$\frac{\partial B_{x}}{\partial x}=\frac{48}{49}\;\sqrt{\frac{3}{7}}\;\frac{\mu_{0}\;N\;I}{R^{2}}$$

This gradient produces the magnetic force responsible for the translational motion of the milligripper.

### 2.3. Simulation of the Milligripper

To determine the two relations (Equations (10) and (11)), the magnets are modeled as two-point magnetic dipoles, and it is assumed that the distance between the magnets is much larger than their size (d≫a). To verify that this assumption is valid for the dimensions of the milligripper, three-dimensional magnetic simulations were performed using the finite element software Altair Flux3D (2025.0.0.1146, Inc., Troy, MI, USA) Additionally, the discrepancies obtained for the repulsive force are less than 4%, while those for the torque remain below approximately 9%, which validates the approximate theoretical relationships. For the cubic magnets used here, the point-dipole approximation is well justified. The cube minimizes the dipole-approximation error among rectangular magnet geometries, with the error falling below one percent within a few magnet sizes [44]; additionally, the separation-to-size ratio of the present device (d≈4.4a) lies well within this range.

### 2.4. Structural Mechanical Modeling

Having validated the magnetic dipole model against finite-element magnetic simulations, the resulting forces and torques were used as mechanical loads in a structural finite-element model using ANSYS Static Structural software (2025 R1, Ansys, Inc., Canonsburg, PA, USA) to predict the opening deformation of the titanium milligripper under actuation. A 2D model of the folded titanium strip with bonded magnets was constructed according to the design geometry described in Section 2. Exploiting the geometric and loading symmetry of the milligripper, only one arm was represented in the simulation, comprising the titanium strip and one NdFeB magnet. The titanium strip (Ti–6Al–4V) was modeled with an isotropic linear elastic law with a Young’s modulus of 113.8 GPa, a Poisson’s ratio of 0.342, and a density of 4430 kg m^−3^, and the yield strength of Ti–6Al–4V is 880 MPa, which defines the upper bound for elastic operation. The NdFeB magnet was assigned linear elastic behavior with a Young’s modulus of 160 GPa, a Poisson’s ratio of 0.24, and a density of 7.5 g cm^−3^, and full bonding was imposed at the magnet–strip interface.

A frictionless support was imposed on the symmetry plane to prevent displacement normal to the symmetry plane, and a point constraint was applied to block rigid body motion along the x-axis. The mesh was generated using quadratic elements with three elements through the strip’s width, yielding an average in-plane element size of approximately 53 µm, with a total of 5584 nodes and 1528 elements. The internal magnet–magnet interaction was represented by a repulsive force $F_{y}$ and a magnetic torque $T_{12}$ acting on the magnet (Equations (10) and (11)). The actuation load under an external magnetic field was represented by a current-dependent torque $T_{\mathrm{open}}\left(I,\alpha \right)$ that was applied as a moment imposed via a remote point rigidly linked to the magnet. The remote point enables the newly calculated magnet angle and magnet distance to the symmetry plane to be measured; both values are required to update the loading values using the software, and the resulting opening and maximum principal stress distribution were computed as a function of the applied current. The following modeling assumptions were adopted throughout: magnets are rigidly bonded to the titanium strip; material properties are homogeneous and isotropic; the components are assumed to be stress-free (internal stresses coming from the manufacturing process not considered); the titanium strip is non-magnetic and does not distort the magnetic field distribution; and dynamic effects are neglected, as the analysis is quasi-static.

#### 2.4.1. Magnet–Magnet Interaction at a Zero External Field

**Figure 3:**
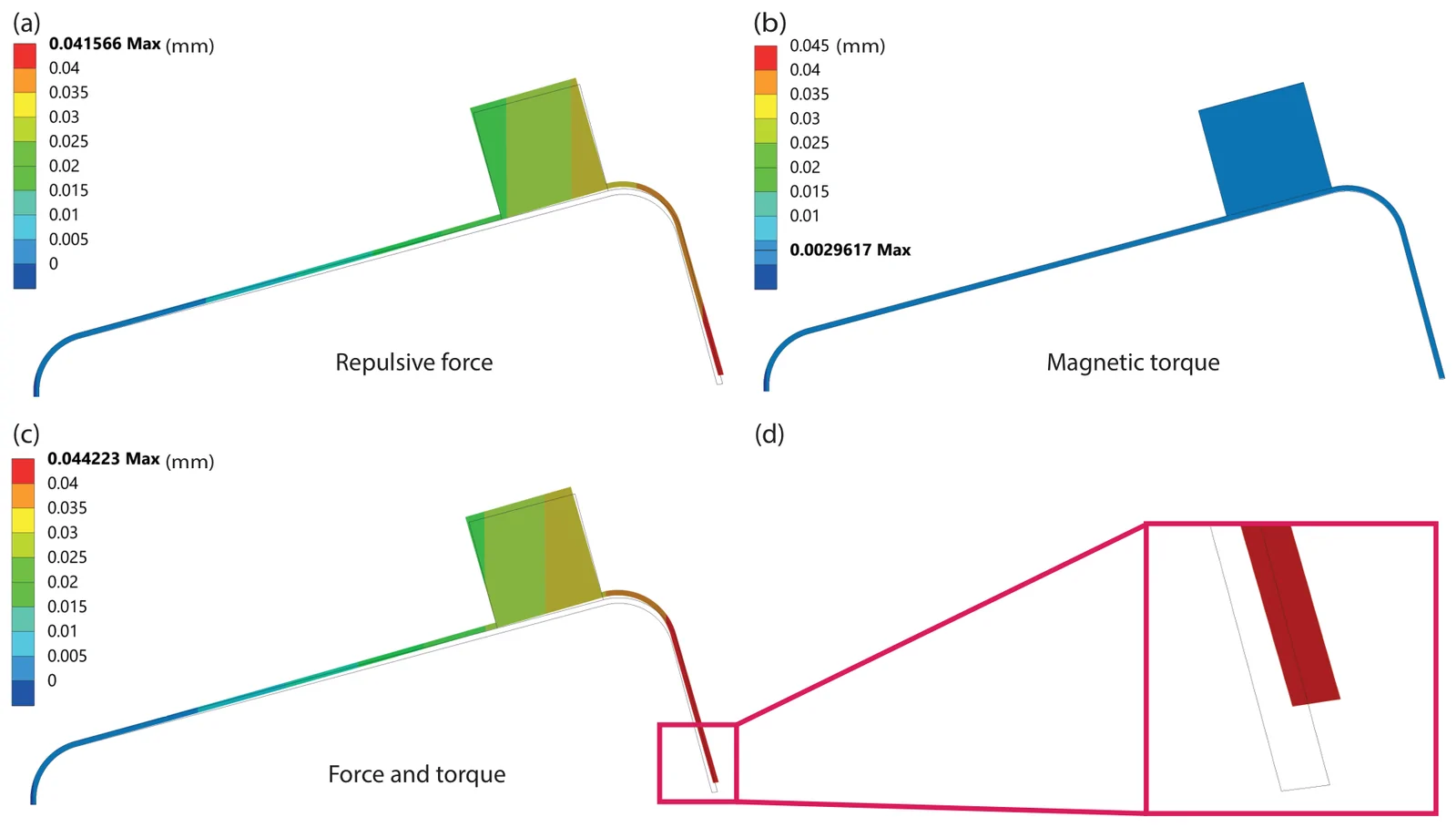
ANSYS 2D finite-element opening contours at a zero external magnetic field. (**a**) Opening under repulsive force only ($F_{y}$). (**b**) Opening under magnetic torque only ($T_{12}$). (**c**) Combined opening under both force and torque ($F_{y}+T_{12}$), with (**d**) a zoom-in view of the tip region; color scale from minimum (blue) to maximum (red).

The internal magnetic interaction between the two permanent magnets was evaluated in the absence of an external field to characterize the zero-field mechanical state of the milligripper. Structural simulations were performed considering three loading cases: repulsive force only ($F_{y}$), magnetic torque only ($T_{12}$), and their combined effect ($F_{y}+T_{12}$). The results are summarized in Table 1, and the corresponding deformation contours are illustrated in Figure 3.

Table 1 shows that the zero-field deformation is very small in all three cases, with the combined opening remaining below 0.05 mm. The internal interaction is dominated by inter-magnet repulsive force: the torque contribution is more than an order of magnitude smaller than the force contribution. The maximum principal stress in the combined case only reaches 3.71 MPa, which is negligible compared to the yield strength of Ti–6Al–4V (880 MPa). These results confirm that the milligripper remains mechanically stable and fully elastic in its normally closed configuration, with an internal magnet–magnet interaction producing no risk of permanent deformation.

#### 2.4.2. Actuation Under an External Magnetic Field

**Figure 4:**
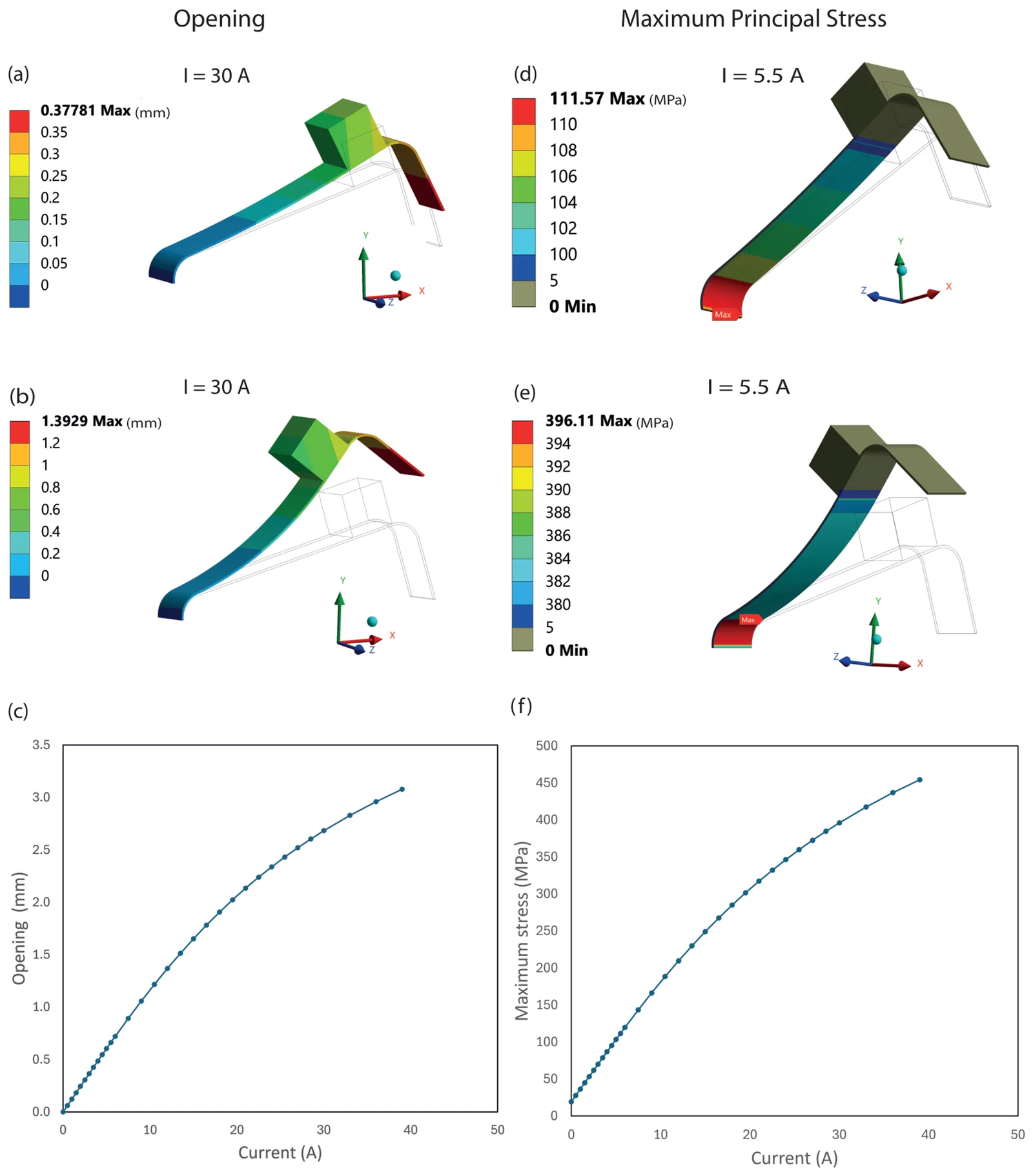
ANSYS finite-element simulation results for milligripper actuation under increasing coil current. Opening contours (left column) and maximum principal stress distributions of one arm (right column) at (**a**,**d**) I=5.5 A and (**b**,**e**) I=30 A. (**c**) Total simulated opening of the milligripper (both arms) relative to the initial assembled configuration and (**f**) maximum principal stress as a function of applied current.

The actuation behavior under an increasing external magnetic field was analyzed by coupling magnetic force and torque computation with structural deformation modeling, with the opening of the milligripper and the maximum principal stress shown in Figure 4.

Figure 4a,b show the simulated deformation contours of one arm at I=5.5 A and I=30 A, respectively, illustrating the progressive bending of the titanium strip as the applied current increases. Figure 4c shows the total opening displacement of the milligripper, which is defined as the tip separation between both arms relative to the initial assembled configuration. The opening increases monotonically with applied current, reaching approximately 0.7 mm at 5.5 A and 2.7 mm at 30 A. The relationship between opening and current remains nearly linear in the low-current regime (1–6 A); in contrast, at higher currents, a progressive deviation from linearity attributed to geometric nonlinearities in the titanium strip as deformation increases and to the reduction in internal magnet–magnet interaction as the arms separate appears.

Figure 4d,e show the maximum principal stress distribution across one arm at I=5.5 A and I=30 A, respectively, while Figure 4f shows its evolution as a function of applied current. The stress distribution evolves smoothly with increasing current: at 5.5 A, the maximum principal stress reaches approximately 112 MPa, rising to 396 MPa at 30 A. Although significant, this value remains well below the yield strength of Ti–6Al–4V (880 MPa). Additionally, the stresses in compression have similar values, confirming that the device operates within the elastic regime even at elevated current levels. The stress distribution is not spatially uniform: the maximum principal stress is concentrated in the region with highest bending, near the base of the arm at the fold, and at the magnet–strip interface and decreases toward the free tip.

### 2.5. Design Trade-Offs

The milligripper geometry is governed by three coupled physical requirements: sufficient orientation torque, adequate translational force, and efficient opening actuation. These requirements impose competing constraints on the key design parameters—magnet volume, inter-magnet separation, inclination angle α, and titanium strip dimensions (thickness and arm length).

**Figure 5:**
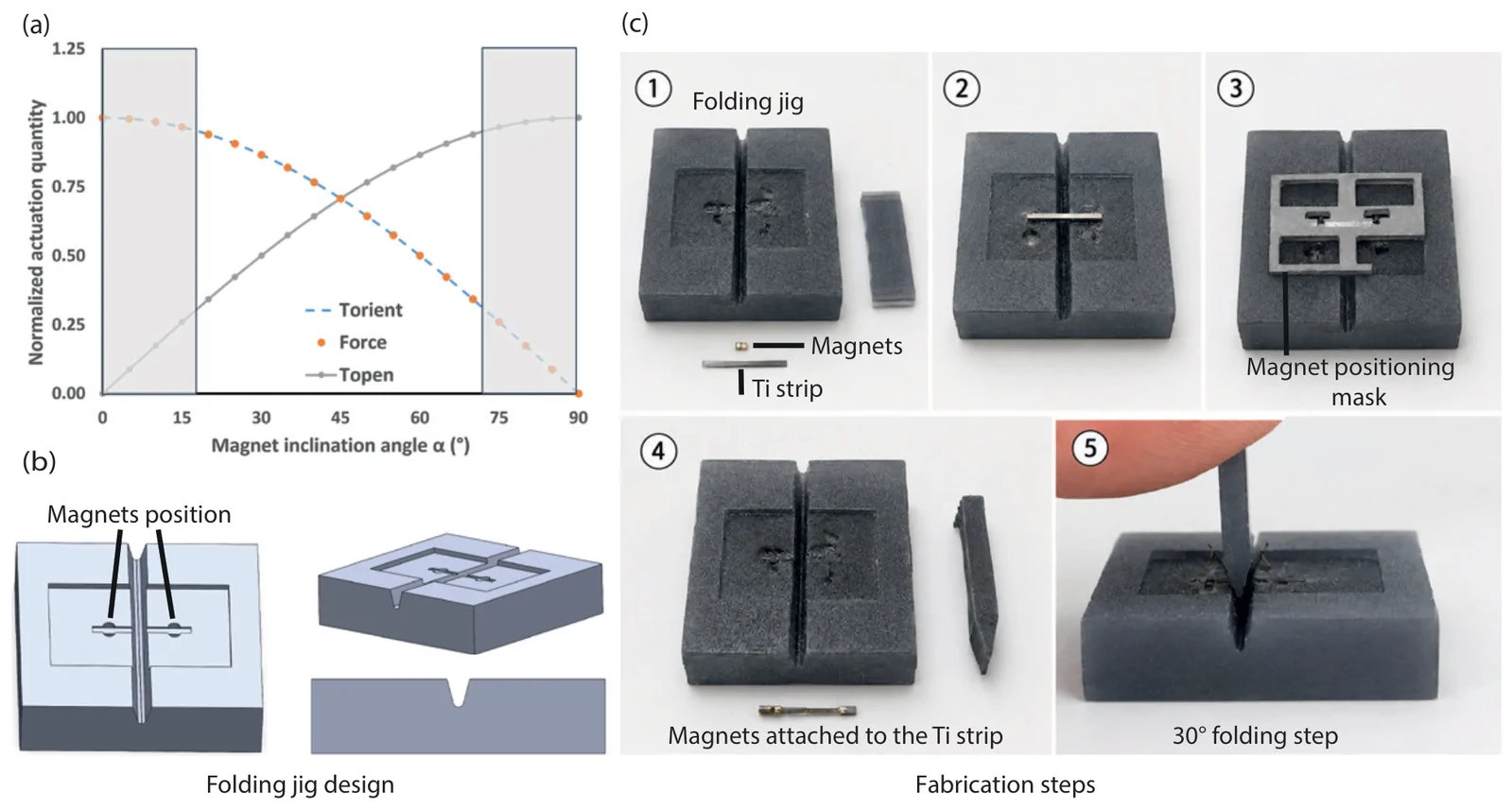
Design trade-off analysis and fabrication of the milligripper. (**a**) Normalized actuation quantities, $T_{\mathrm{orient}}$, $T_{\mathrm{open}}$, and *F* as a function of the magnet inclination angle α. (**b**) Design of the 3D-printed folding jig used to shape the titanium structure and to define the magnet positions. (**c**) Fabrication steps: (1) folding jig, Ti–6Al–4V strip, and NdFeB magnets; (2) positioning of the Ti strip on the jig; (3) alignment of the magnets using a dedicated positioning mask; (4) magnets bonded to the Ti strip; (5) folding of the structure to its final geometry using the jig groove.

The inclination angle α plays the central role in balancing the three actuation requirements, as illustrated in Figure 5a. The orientation torque $T_{\mathrm{orient}}$ and the translational force *F* scale with cosα, while the opening torque $T_{\mathrm{open}}$ scales with sinα. If α is too small, the torque component responsible for bending becomes insufficient to open the arms. Conversely, as α approaches $90^{\circ }$, the magnetic moments of the two magnets become nearly antiparallel, reducing the resultant moment to $m_{\mathrm{tot}}\approx 0$ and severely limiting orientation and displacement capability. Values of α outside the range $\left[15^{\circ },\;75^{\circ }\right]$ result in at least one actuation component falling below 25% of its maximum (shaded regions in Figure 5a). An angle of $45^{\circ }$ would distribute the contributions equally; however, since the field required to open the gripper exceeds that required for orientation and translation—the gripper can be positioned and oriented within the homogeneous field without deforming—a larger angle is preferred. The value $\alpha =75^{\circ }$ was therefore selected to favor $T_{\mathrm{open}}$ while preserving an adequate resultant dipole moment and avoiding excessively thin or long titanium strips that would compromise structural robustness.

The remaining geometric parameters follow similar trade-offs. Larger magnets increase actuation torque but amplify internal magnet–magnet coupling and structural stress. Additionally, increasing the inter-magnet separation reduces internal repulsion but lowers structural stiffness. The titanium strip thickness directly governs arm stiffness: if the strip is too thick, the elastic restoring force prevents opening under the available magnetic torque; if it is too thin, the applied stress exceeds the elastic limit of Ti–6Al–4V, causing permanent deformation. A longer arm length facilitates opening by reducing bending stiffness and increasing the moment arm, thereby amplifying tip displacement for a given actuation force.

The selected geometry—$\alpha =75^{\circ }$, the titanium strip dimensions, and inter-magnet separation d=2.22 mm—satisfies all three requirements simultaneously, ensuring sufficient opening torque, adequate translational and rotational capability, and stresses within the elastic regime of Ti–6Al–4V. Moreover, this geometry was determined from coupled physical constraints rather than empirical adjustment, ensuring predictable and scalable magneto-mechanical behavior.

## 3. Experimental Fabrication and Setup

### 3.1. Milligripper Fabrication

**Figure 6:**
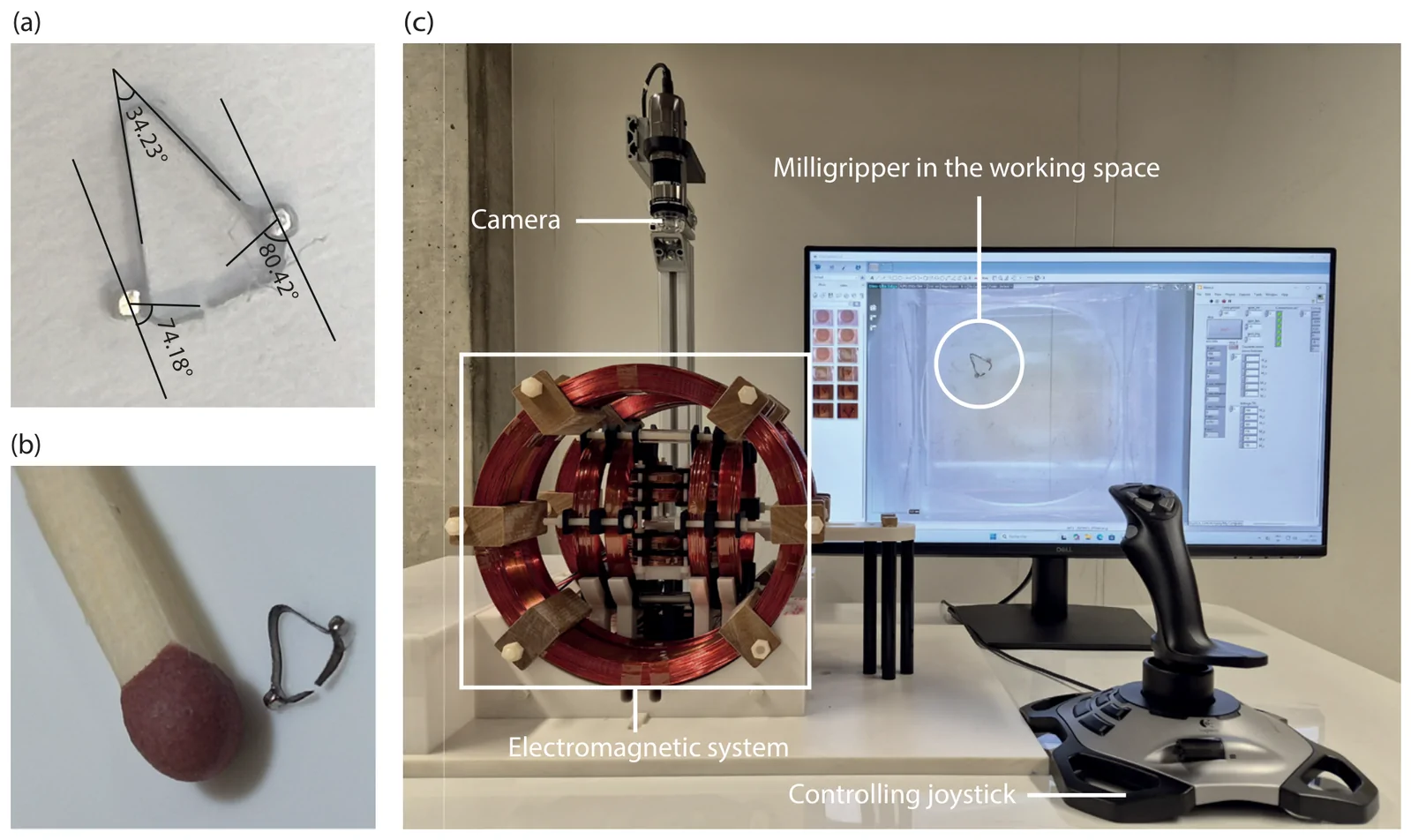
Fabricated milligripper and experimental platform. (**a**) Optical measurement of the magnet inclination angles on a fabricated device. All angles correspond to in-plane projections measured from top-view images. (**b**) Photograph of the fabricated milligripper next to a matchstick head, illustrating its millimeter-scale dimensions. (**c**) Overview of the three-axis Helmholtz–Maxwell electromagnetic coil system with the milligripper positioned in the central working space (monitored by an overhead optical camera). The joystick-based control interface and real-time display are visible in the background.

The milligripper body was fabricated from a Ti–6Al–4V strip by laser cutting, followed by jig-assisted folding and magnet assembly. Titanium was selected due to its high strength-to-weight ratio, corrosion resistance, and excellent biocompatibility, characteristics that make it suitable for potential biomedical applications. The cutting process was performed using a LaserPecker LP5 (Shenzhen Hingin Technology Co., Ltd, Shenzhen, China) fiber laser system (20 W output power, 1064 nm wavelength, and 25 kHz repetition frequency), and the laser parameters were adjusted to precisely cut the thin titanium strip (8.0×0.5×0.03 mm^3^) while minimizing thermal deformation and edge roughness. The subsequent assembly and folding steps were performed using a dedicated folding jig (Figure 5b,c). The jig integrates a central groove that defines the folding line and reference features that define the positions of the magnets on the strip. The alignment mask on the folding jig constrains the location of the magnets along the titanium arms, and the inclination itself is then fixed geometrically by the fold. Because the two arms are symmetric along the fold line, folding the strip to the nominal 30° opening angle constrains each magnet to the design inclination of $\alpha =75^{\circ }$ by construction. After laser cutting, the titanium strip was placed on the jig, and two 500 μm cubic NdFeB permanent magnets were aligned on its outer surfaces using a dedicated positioning mask and bonded with a small amount of epoxy adhesive to ensure rigid attachment. The titanium strip was then manually folded along the jig groove to a nominal opening angle of 30° (±5°), forming the final milligripper geometry. The folding process creates a normally closed configuration in which the two gripper arms converge at the tip, allowing elastic opening during actuation. The resulting device is shown in Figure 6b alongside a matchstick head, illustrating its millimeter-scale dimensions.

The magnet inclination angle obtained after assembly was verified optically on the fabricated devices (Figure 6a). Specifically, the measured inclination angles of 74.18° and 80.42° confirm that the fold-defined geometry reproduces the nominal design value of $\alpha =75^{\circ }$ within a tolerance of approximately $\pm 5^{\circ }$, which was inherited directly from the $30^{\circ }\pm 5^{\circ }$ tolerance of the manual folding step. According to the parametric analysis presented in Section 2, deviations of this magnitude have a limited effect on the resulting actuation torque, since the opening torque varies slowly in the vicinity of $\alpha =75^{\circ }$.

### 3.2. Magnetic Actuation System

Magnetic actuation was achieved using a three-axis Helmholtz–Maxwell coil system comprising three Helmholtz coil pairs and three Maxwell coil pairs aligned along the x-, y-, and z-axes, generating both uniform fields and controlled gradients (Figure 6b), with he physical parameters of the coil system summarized in Table 2. Assuming the milligripper behaves as a magnetic dipole, the system provides control over three translational degrees of freedom (force) and two rotational degrees of freedom (torque), as it cannot rotate along the axis of the resultant magnetic moment ${\vec{m}}_{\mathrm{tot}}$.

The working area (Figure 6) used for the experiments corresponds to the central region of the coil system (35×35×25 mm^3^), where the magnetic field distribution is sufficiently uniform to ensure reproducible actuation behavior. The electromagnetic actuation system was coupled to a real-time control interface that allows intuitive manipulation of the magnetic fields (see Figure 6). Coil currents were controlled through a joystick-based interface, thus enabling the operator to adjust the magnetic field direction and amplitude interactively. This setup enables the orientation, translational motion, and opening of the milligripper within the workspace to be easily controlled. The experimental platform provides a user-friendly environment for magnetic micromanipulation experiments.

### 3.3. Imaging and Measurement

The actuation behavior of the milligripper was characterized using optical microscopy combined with digital video recording, as shown in Figure 6. The opening displacement of the gripper tips and the rotation angle of each arm were measured as a function of the applied current in the Helmholtz coils, with measurements performed under quasi-static conditions to ensure that the structure reached mechanical equilibrium at each current step.

**Figure 7:**
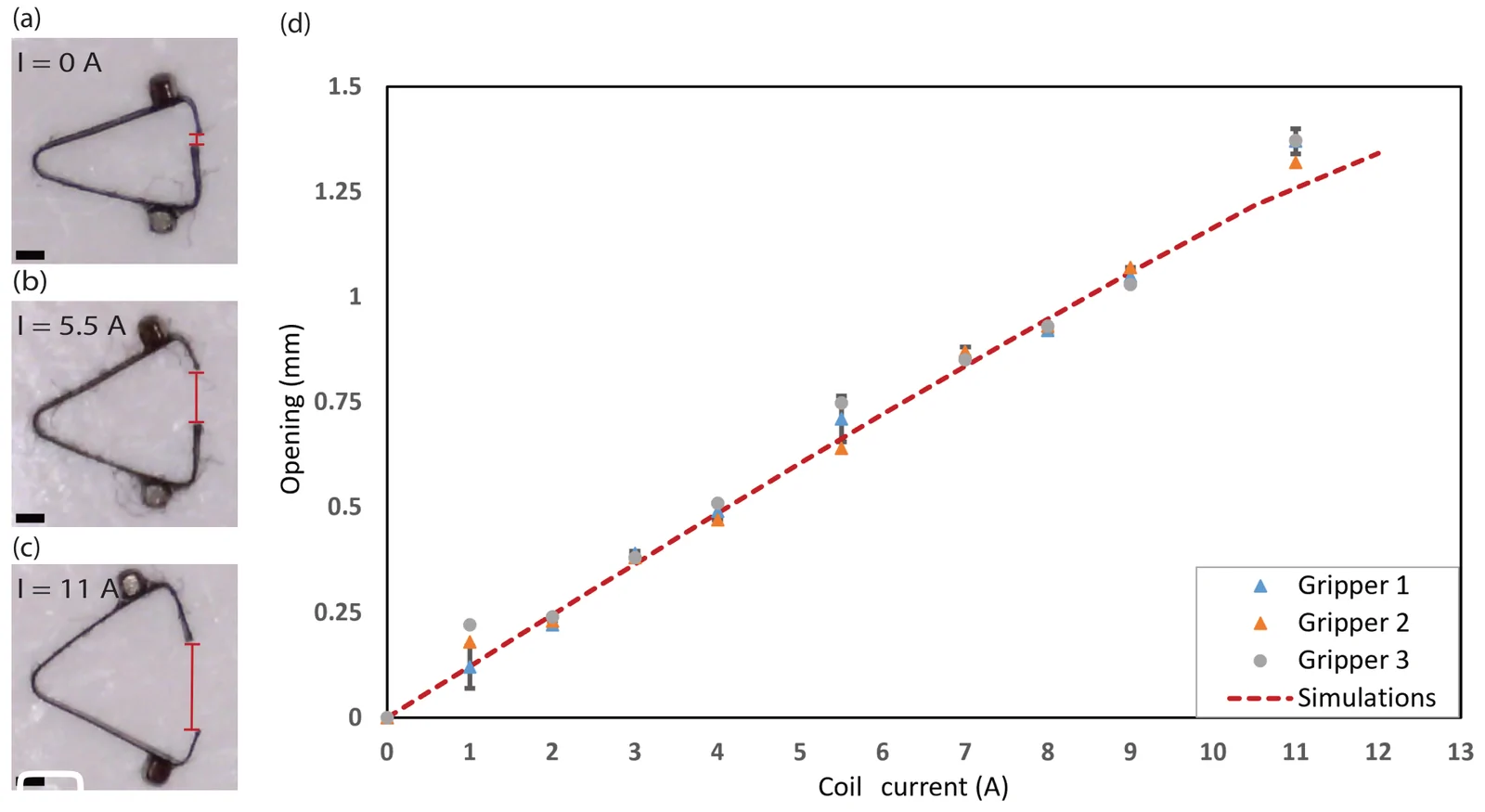
Experimental characterization of milligripper opening in water under increasing coil current. Optical microscopy images of the milligripper at (**a**) I=0 A, (**b**) I=5.5 A, and (**c**) I=11 A. Red markers indicate the measured tip separation. Scale bar: 500 μm. (**d**) Opening of the milligripper relative to the initial assembled configuration as a function of applied current, measured in water on three fabricated devices (Grippers 1–3), compared with finite-element simulation predictions (red dashed line). Error bars indicate the standard deviation over n=3 devices.

Experiments were conducted for current values ranging from 0 A to 11 A. For each current level, the milligripper opening was recorded, and the corresponding opening displacement was measured from the captured images, as shown in Figure 7a–c. The measured experimental values were subsequently compared with finite-element simulations to validate the proposed magneto-mechanical model.

## 4. Experimental Results and Discussion

### 4.1. Experimental Validation of Milligripper Actuation

Table 3 summarizes the average measured total opening displacement of three devices in water as a function of the applied current in the Helmholtz coils. Figure 7a–c present optical images of the milligripper’s total opening under different actuation currents (0 A, 5.5 A, and 11 A). In the absence of an external magnetic field, the milligripper has an opening displacement of approximately 0.3 mm due to the combined effect of structural elasticity, manual fabrication limitations, and internal magnet–magnet interaction.

As the current increases, the external magnetic field generates torque on each magnet, producing a bending moment on the titanium arms. This torque gradually opens the gripper, resulting in an increase in the separation between the tips.

Figure 7d shows the opening of the milligripper relative to the initial assembled configuration as a function of the applied coil current, as measured in water using three independently fabricated devices. The opening increases monotonically with current, from a resting tip separation of 0.306 mm at I=0 A to an average opening of 1.372 mm at I=11 A relative to the initial assembled configuration, corresponding to a magnetically induced opening of 1.678 mm, as shown in Table 3. The measurements are in good agreement with the finite-element simulation predictions across the full current range, and the consistent behavior of the three devices demonstrates the repeatability of the fabrication process and the actuation response, with the error bars indicating the standard deviation across the three devices.

To further assess repeatability under repeated actuation, a representative device was subjected to five successive open–close cycles at a fixed current of 5.5 A, with the measured opening displacements summarized in Table 4. The opening remains consistent across cycles, with a mean of 1.02 mm and a standard deviation of 0.09 mm, in agreement with the value reported in Table 3 at the same current. Notably, the opening fluctuates about the mean without any systematic downward trend, and the device returns to its closed configuration after each cycle, indicating that no permanent deformation accumulates under repeated use. This behavior is consistent with the finite-element analysis, which predicts maximum principal stresses well below the yield strength of Ti–6Al–4V (880 MPa), confirming that the milligripper operates in a fully elastic and repeatable regime.

Minor discrepancies between the simulation and experiment can be attributed to several factors, including fabrication tolerances, variations in the thickness of the adhesive layer, internal stresses due to manufacturing steps, slight magnet misalignment, and friction effects not included in the idealized model. Additionally, small non-uniformities in the magnetic field distribution and measurement uncertainties may contribute to minor deviations.

The agreement between analytical predictions, finite-element simulations, and experimental measurements confirms the validity of the proposed magneto-mechanical model, and the results demonstrate that the actuation mechanism is accurately captured by the dipole-based framework and that the milligripper operates in a predictable and controllable manner.

### 4.2. Controlled Orientation, Opening, Closing, and Motion

**Figure 8:**
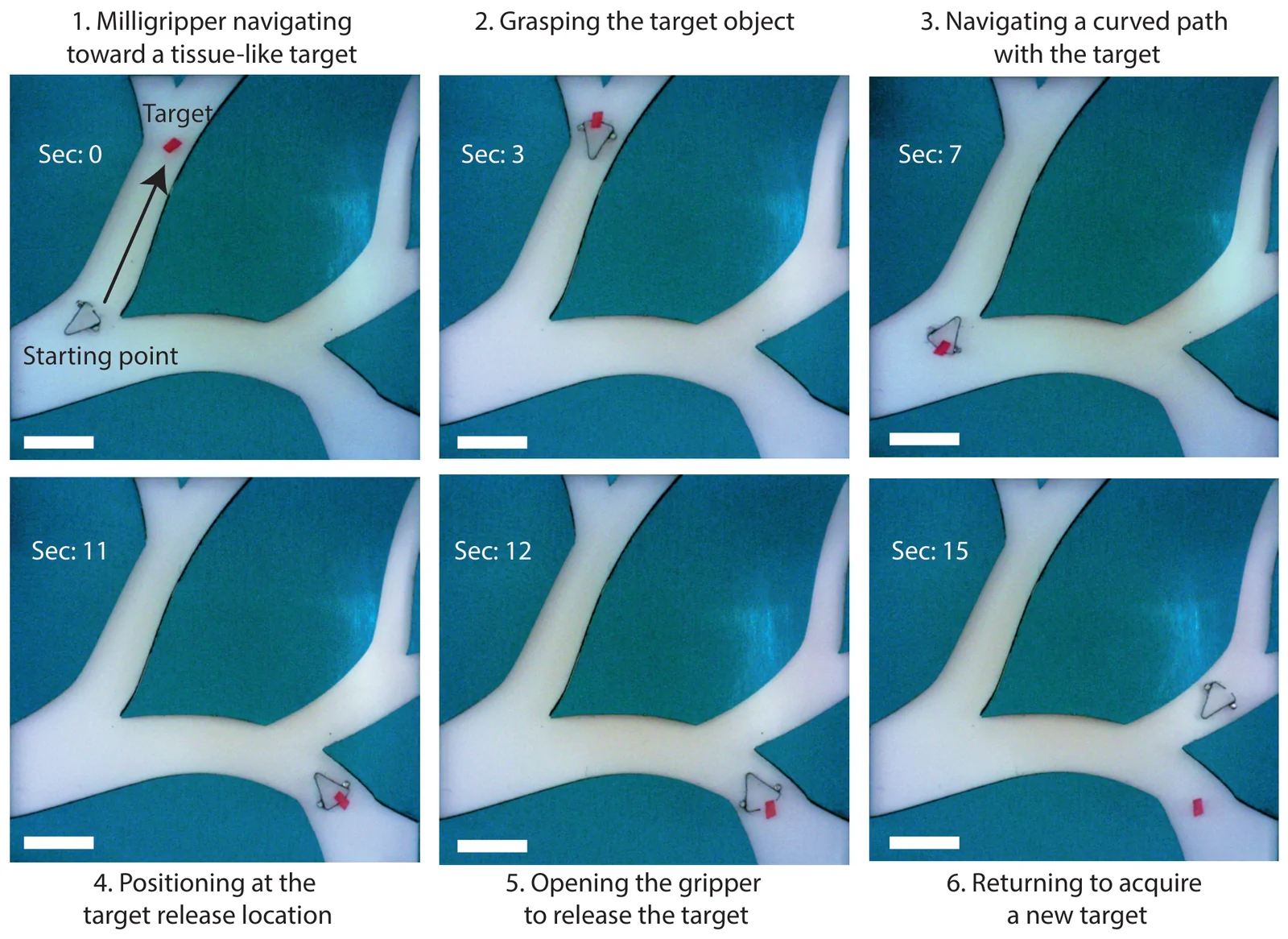
Sequential demonstration of a complete pick-and-place cycle performed by the magnetic milligripper in a vascular-inspired phantom. The target is a tissue-like PEGDA hydrogel bead, and the time stamps in each frame indicate the elapsed time from the start of the sequence. Scale bar: 5 mm.

To demonstrate the functional capability of the milligripper, a complete pick-and-place cycle was performed in a vascular-inspired phantom using a tissue-like PEGDA hydrogel bead (3% *w*/*v* in water, roughly rectangular, and ≈1 mm in its longest dimension) as the target (Figure 8). For optical visibility, the PEGDA bead was stained by immersing it in a drop of red ink prior to the experiment. Additionally, the vascular-inspired phantom consisted of branched channels with a depth of approximately 0.5 mm, which is comparable to the thickness of the milligripper body (≈0.5 mm envelope), so that the device was confined to near-planar motion within the channel. The milligripper was navigated from its starting position toward the target, which was grasped within 3 s. The gripper then transported the bead along a curved path through the branched channels and positioned it at the release location within 11 s from the start of the sequence. The target was released at t=12 s after controlled opening of the gripper, after which it returned toward the acquisition area for a subsequent pick-up cycle, thus completing the full sequence in 15 s.

This demonstration confirms that the milligripper can grasp, transport through branched geometries, and controllably release a soft, tissue-like object without visible damage to the hydrogel bead. While this demonstration (Figure 8) was performed in air, the opening behavior of the milligripper was characterized in water (Figure 7d), confirming that the actuation principle operates in liquid environments.

Beyond the demonstrated pick-and-place capability in a vascular-inspired phantom, the milligripper architecture is directly applicable to a broader range of laboratory and biomedical scenarios. In a biological laboratory context, the platform can be used to manipulate soft tissue samples, cell clusters, or microorganisms in fluidic environments, where contactless, precise grasping without mechanical damage to the specimen is required. From a clinical perspective, the milligripper can be integrated at the distal tip of a magnetically steered catheter, thus enabling simultaneous navigation and gripping within confined anatomical passages such as vascular or gastrointestinal channels. In both cases, the same external coil system controls orientation, translation, and opening through a single unified magnetic field, without requiring hardware reconfiguration.

### 4.3. Localization

A key remaining challenge for deploying magnetic milligrippers in clinical and laboratory environments is real-time localization within opaque or confined spaces, where optical tracking suffers from line-of-sight constraints. To address this, an electrical impedance-based localization approach was developed and experimentally validated on the present milligripper platform.

The method relies on a multiplexed planar electrode array surrounding a saline-filled sensing chamber, in which the conductive Ti–6Al–4V body of the milligripper induces localized impedance perturbations that shift systematically with its position. Impedance measurement enables non-invasive microrobot localization by detecting conductivity variations from boundary measurements [45] and has recently demonstrated potential for real-time milligripper tracking [46].

**Figure 9:**
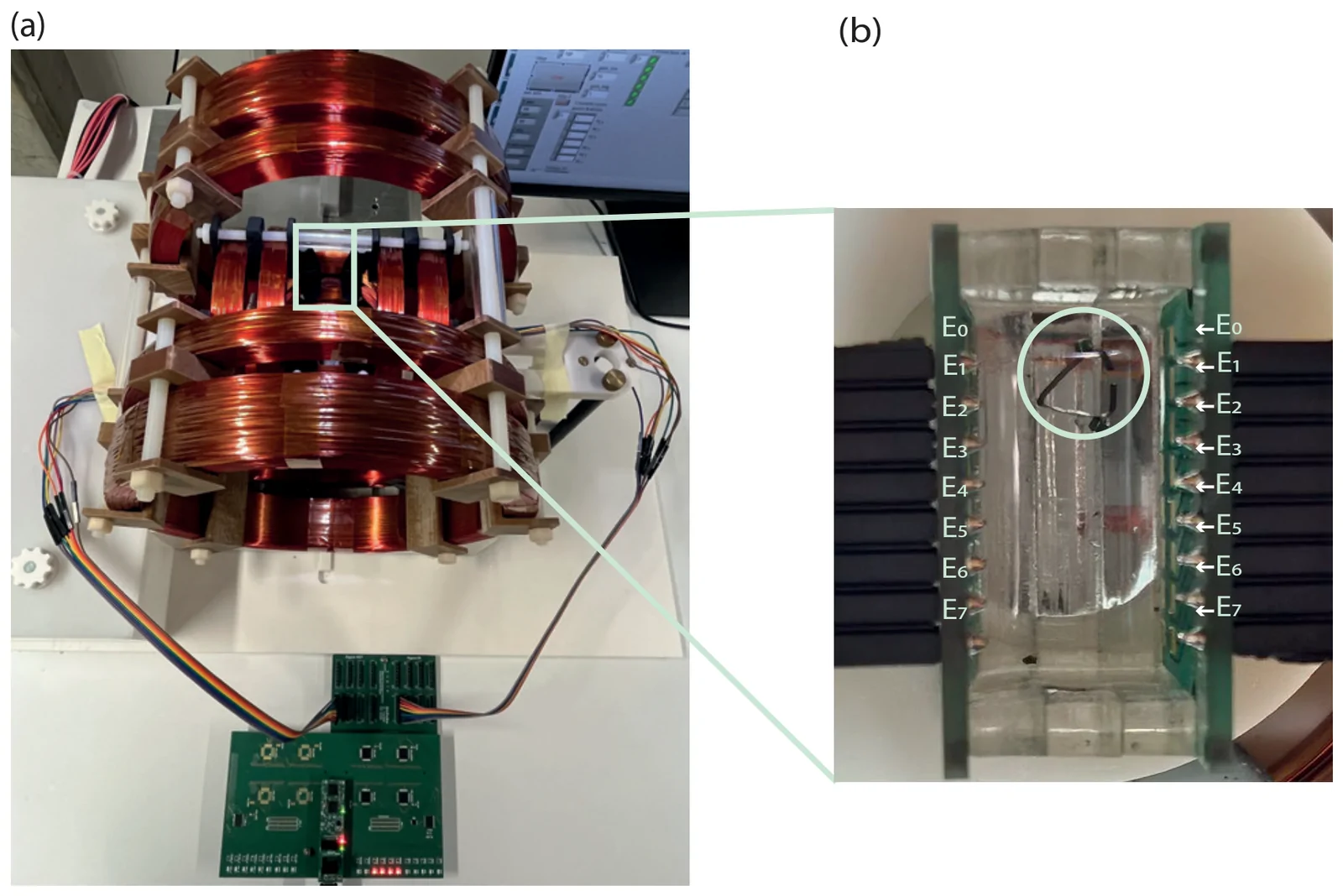
Impedance-based localization setup integrated with the milligripper platform. (**a**) Overview of the laboratory platform combined with the impedance measurement system; the sensing chamber is positioned at the center of the milligripper workspace, while the multiplexing electronics are located adjacent to it. (**b**) Zoomed-in view of the 15 mm sensing chamber with the milligripper; electrode pair locations used for impedance measurements are indicated.

A custom sensing chamber was developed, comprising two parallel plates with several rows of eight planar electrodes with an area of 4 mm^2^ arranged in opposing pairs. The chamber length is 30 mm, with an inter-plate distance of 10 mm or 15 mm, and is filled with a 0.9% NaCl solution. Impedance measurements are performed using analogue multiplexers controlled via I^2^C and dedicated front-end electronics that generate AC excitation with synchronous acquisition and operate in the 40–60 kHz frequency range to limit capacitive effects at the electrode–electrolyte interface. The measured impedance variation is defined as

(14) $$\Delta Z=\frac{|Z-Z_{0}|}{Z_{0}}\times 100$$

where $Z_{0}$ is the baseline impedance measured in the absence of the milligripper and *Z* is the impedance measured with the milligripper present in the chamber. The experimental setup is illustrated in Figure 9, showing the sensing chamber positioned at the center of the milligripper platform, with the multiplexing and measurement electronics located adjacent to it.

Importantly, it was experimentally verified that the magnetic fields used for milligripper actuation do not influence the impedance measurements and, conversely, that the impedance sensing system does not perturb the magnetic control of the milligripper—a critical requirement for simultaneous localization and actuation in practice.

Preliminary results demonstrate that the milligripper position can be reliably identified by monitoring the impedance variation across electrode pairs. As summarized in Table 5, variations of 2–3% were observed at electrodes nearest to the milligripper position (E2), while adjacent electrodes and distant electrodes exhibited variations of 1–2% and variations below 1%, respectively, for both 10 mm and 15 mm chamber widths. Real-time tracking during controlled displacement was also demonstrated experimentally. A full characterization of this localization approach, including sensing architecture optimization and the acquisition strategy, will be the subject of a dedicated future publication.

## 5. Conclusions

In summary, we present the design, modeling, and experimental validation of a rigid, normally closed magnetic milligripper based on a folded Ti–6Al–4V structure with two symmetrically inclined NdFeB permanent magnets actuated through magnetic torque-induced elastic bending. Finite-element simulations and experimental measurements confirmed a fully elastic and reproducible opening response across the investigated actuation range, in close agreement with the analytical dipole model. Additionally, a complete pick-and-place cycle—grasping, transport through a branched vascular-inspired phantom, and controlled release of a soft-tissue-like target—was further demonstrated, illustrating the functional manipulation capability of the platform.

A key practical advantage of the platform is the simplicity of its control: orientation, opening/closing, and translation require no automated control algorithms. The architecture provides a structurally simple, analytically tractable, and scalable platform for magnetic manipulation at the millimeter scale. Complementing the actuation capability, an impedance-based localization approach was developed and validated on the same platform, enabling real-time milligripper tracking in conductive environments without perturbing the magnetic control.

The present study constitutes a proof-of-concept demonstration under controlled laboratory conditions. Before clinical translation, several practical constraints must be addressed: biocompatible encapsulation of the NdFeB magnets and epoxy adhesive, mechanical durability of the bonded magnet–strip interface under repeated actuation cycles, sterilization, and corrosion resistance and thrombogenicity in physiological fluids. Additionally, hydrodynamic drag under blood flow and tissue friction, which were not present in the optically accessible phantom used here, needs to be accounted for prior to operation in realistic physiological environments. Lastly, localization in optically opaque tissue poses an additional open challenge.

Future work will focus on extending the platform toward biomedical use, including the development of a miniaturized, potentially biodegradable version of the milligripper. Thus, ongoing research aims to identify suitable biocompatible materials for the gripper structure and magnetic particles capable of safe degradation within the body after completing their task. Such transient microrobotic tools could enable minimally invasive interventions without the need for retrieval.

## Figures and Tables

**Table 1:** Influence of internal magnetic repulsion force $F_{y}$ and magnet–magnet torque $T_{12}$ on milligripper opening at a zero external field.

Quantity	Unit	Force Only	Torque Only	Force + Torque
		**($F_{y}$)**	**($T_{12}$)**	**($F_{y}+T_{12}$)**
Opening	mm	0.041	0.003	0.044
Maximum stress	MPa	3.5	0.3	3.71

**Table 2:** Physical parameters of the three-axis Helmholtz–Maxwell coil system used for milligripper actuation.

Parameter	Unit	Helmholtz Coils			Maxwell Coils		
		X	Y	Z	X	Y	Z
Mean radius	mm	68	117	42	68	117	42
Turns per coil	–	560	945	350	636	2035	270
Wire diameter	mm	0.6	0.6	0.6	0.6	0.6	0.6

**Table 3:** Experimental average total opening of the milligripper in water as a function of the coil current.

Current (A)	Magnetic Field (mT)	Total Opening (mm)
0	0	0.306
5.5	39.05	1.054
11	78.2	1.678

**Table 4:** Repeatability of the milligripper opening over five successive open–close cycles at a fixed actuation current of 5.5 A.

Cycle	1	2	3	4	5	Mean ± SD
Total Opening (mm)	1.054	0.950	1.120	1.068	0.900	1.02 ± 0.09

**Table 5:** Experimental relative impedance variation (ΔZ) measured for each electrode pair with the milligripper positioned at the E2 electrode location for chamber widths of 10 mm and 15 mm.

Electrode Pair	E0	E1	E2	E3	E4	E5	E6	E7
Width: 10 mm, ΔZ [%]	2	3	2	1	1	1	0	0
Width: 15 mm, ΔZ [%]	2	3	2	1	0	0	0	0

## Data Availability

The data presented in this study are available on request from the corresponding author due to proprietary considerations and ongoing research related to the further development of the technology.
